# 15-Epi-LXA_4_ and MaR1 counter inflammation in stromal cells from patients with Achilles tendinopathy and rupture

**DOI:** 10.1096/fj.201900196R

**Published:** 2019-03-27

**Authors:** Stephanie G. Dakin, Romain A. Colas, Julia Newton, Stephen Gwilym, Natasha Jones, Hamish A. B. Reid, Simon Wood, Louise Appleton, Kim Wheway, Bridget Watkins, Jesmond Dalli, Andrew J. Carr

**Affiliations:** * Nuffield Department of Orthopaedics, Rheumatology, and Musculoskeletal Sciences, Botnar Research Centre, Nuffield Orthopaedic Center, University of Oxford, Oxford, United Kingdom;; † Lipid Mediator Unit, William Harvey Research Institute, Barts and the London School of Medicine and Dentistry, Queen Mary University of London, London, United Kingdom;; ‡ Centre for Inflammation and Therapeutic Innovation, Queen Mary University of London, London, United Kingdom

**Keywords:** resolution, tendon, 15-epi-LipoxinA_4_, maresin-1

## Abstract

Resolution of inflammation is poorly understood in Achilles tendon disorders. Herein, we investigated the bioactive lipid mediator profiles of tendon-derived stromal cells isolated from patients with Achilles tendinopathy (AT) or Achilles rupture (AR) under baseline and IL-1β–stimulated conditions. We also determined whether incubating these cells with 2 of the mediators produced by tendon-derived stromal cells, 15-epi-Lipoxin A_4_ (15-epi-LXA_4_) or maresin (MaR)-1, moderated their proinflammatory phenotype. Under baseline conditions, AT cells showed concurrent increased levels of proinflammatory eicosanoids and proresolving mediators compared with AR cells. IL-1β treatment induced profound prostaglandin E_2_ release in AR compared with AT cells. Incubation of IL-1β treated AT and AR tendon-derived stromal cells in 15-epi-LXA_4_ or MaR1 reduced proinflammatory eicosanoids and potentiated the release of proresolving mediators. These mediators also induced specialized proresolving mediator (SPM) biosynthetic enzymes arachidonate lipoxygenase (ALOX) 12 and ALOX15 and up-regulated the proresolving receptor ALX compared with vehicle-treated cells. Incubation in 15-epi-LXA_4_ or MaR1 also moderated the proinflammatory phenotype of AT and AR cells, regulating podoplanin, CD90, signal transducer and activator of transcription (STAT)-1, IL-6, IFN regulatory factor (IRF) 5, and TLR4 and suppressed c-Jun N-terminal kinase 1/2/3, Lyn, STAT-3, and STAT-6 phosphokinase signaling. In summary, we identify proresolving mediators that are active in AT and AR and propose SPMs, including 15-epi-LXA_4_ or MaR1, as a potential strategy to counterregulate inflammatory processes in these cells.—Dakin, S. G., Colas, R. A., Newton, J., Gwilym, S., Jones, N., Reid, H. A. B., Wood, S., Appleton, L., Wheway, K., Watkins, B., Dalli, J., Carr, A. J. 15-Epi-LXA_4_ and MaR1 counter inflammation in stromal cells from patients with Achilles tendinopathy and rupture.

Achilles tendinopathy (AT) and Achilles rupture (AR) are common causes of pain and disability affecting athletes and nonathletic patients (1). These injuries require prolonged rehabilitation and have high rates of recurrent injury, and their pathology is frequently bilateral (2, 3). Current advocated therapies for AT include a standard 3-mo eccentric training program (4) and nonsteroidal anti-inflammatory drugs (NSAIDs) (5, 6). Patients with persistent tendinopathy are treated with a variety of therapies, including dry needling, local infiltration of platelet-rich plasma, high-volume injection (HVI), glyceryl trinitrate, or surgery (7–11). The lack of high-quality, multicenter, randomized placebo-controlled clinical trials hinders the ability to determine which, if any, of these therapies are clinically effective. Of importance, many of these therapies do not address the pathobiology associated with AT. Furthermore, cyclooxygenase 2–selective NSAIDs dampen protective responses regulating the resolution of inflammation (12, 13), paradoxically impeding the ability of inflamed tendons to heal. Some patients develop AR and are managed surgically or conservatively with immobilization and subsequent physical therapy (14). A recent meta-analysis showed that operative treatment reduced the risk of rerupture compared with nonoperative treatment, highlighting that management should be tailored toward patient-specific factors and shared decision making (15).

The etiology of Achilles tendon disease is complex and multifactorial and includes the effects of exercise overuse, aging, and genetic factors (16–18). The importance and role of inflammation in the disease of these energy-storing tendons has been highly debated, and historically, the disease was frequently described as degenerative (19, 20). More recent studies highlight a paradigm shift toward improved understanding of inflammatory processes in Achilles tendon disorders whereby immune-competent cells including macrophages, T cells, NK, and mast cells have been identified in patient biopsy specimens (21, 22). We recently identified chronic inflammation as a feature of midportion AT and AR. Tendinopathic and ruptured Achilles showed complex tissue inflammation signatures involving NF-κB, IFN, and signal transducer and activator of transcription (STAT)-6 activation pathways. IFN markers IFN regulatory factor (IRF) 1 and IRF5 were highly expressed in tendinopathic samples, whereas ruptures showed increased prostaglandin-endoperoxide synthase 2 and *IL-8* mRNA expression (22). These differences in proinflammatory profiles were attributed to acute inflammation and increased vascularization arising from the event of traumatic rupture. Of importance, resident stromal fibroblasts (tendon-derived stromal cells), which constitute the majority cell type in Achilles tendons, and their roles in sustaining chronic inflammation remain underinvestigated. We recently identified tissues and cells isolated from patients with tendinopathic and ruptured Achilles that showed a proinflammatory phenotype, expressing markers of stromal fibroblast activation including podoplanin (PDPN) and CD106 (VCAM-1) (22). In functionally distinct positional tendons, we also identified tendon-derived stromal cells from patients with shoulder tendon tears that showed dysregulated resolution responses compared with cells isolated from the tendons of healthy volunteers (23).

To date, resolution of inflammation has not been investigated in Achilles tendon disorders. The current study was performed to advance understanding of resolution processes active in human AT and AR. This study has 2 main objectives: first, to investigate if tendon stromal cells isolated from patients with AT and AR show distinct bioactive lipid mediator (LM) profiles in order to assess whether inflammation-resolution mechanisms are differentially activated in these distinct conditions; and second, to identify new therapeutic approaches to counter inflammation and potentiate resolution in patient-derived Achilles tendon stromal cells. We demonstrate that cells isolated from patients with AT and AR show distinct bioactive LM profiles. Through experiments with representative specialized proresolving mediators (SPMs) 15-epi-Lipoxin A_4_ (15-epi-LXA_4_) and maresin (MaR) 1, we provide evidence that these SPMs regulate the proinflammatory phenotype and promote resolution responses in patient-derived Achilles tendon stromal cells.

## MATERIALS AND METHODS

### Study approval

Tissues from patients with AT or AR were collected under the Office for Research Ethics Committees from Northern Ireland Research Ethics Committee (reference 14/NI/1063). Full informed consent according to the Declaration of Helsinki was obtained from all patients.

### Collection of patient Achilles tendon tissues

Patients with AT were recruited from a sports medicine clinic (*n* = 15). This group comprised 7 female and 8 male patients aged between 41 and 65 yr. Patients presenting to the sports clinic had been symptomatic for several months and failed a standard 3-mo eccentric training program for AT. Ultrasonography was performed to confirm a diagnosis of AT. Patients completed the Victorian Institute of Sports Assessment–Achilles scoring system (24), a validated clinical outcome measure scoring from 0 (severe disease) to 100 (normal function). AT biopsies were collected from patients that presented for HVI, as this procedure represents the next step in standard treatment after failed response to physical therapy. This procedure involves injecting 10 ml 0.5% bupivacaine and 30 ml saline into the pre-Achilles space. AT biopsies were obtained *via* percutaneous ultrasound-guided biopsy under local anesthesia prior to HVI by inserting a 14-gauge Tru-Cut biopsy needle (Merit Medical Systems, South Jordan, UT, USA) into the diseased midportion of the Achilles. This validated biopsy technique is previously described by Dakin *et al*. (22). Tissue biopsy samples were also collected from 15 patients recruited from a trauma unit with acute traumatic AR. This group comprised 4 female and 11 male patients aged between 25 and 60 yr. Tissue biopsy samples were collected up to 48 h after tendon rupture. Full informed consent according to the Declaration of Helsinki was obtained from all patients participating in the study. Study exclusion criteria included previous corticosteroid, platelet-rich plasma, and stem cell intratendinous injections, extracorporeal shockwave therapy, or systemic steroid or methotrexate treatments. Patients who were diabetic and those receiving systemic anticoagulant therapy were also excluded from the study. AT patients did not receive anti-inflammatory medications during their rehabilitation or at the time of tendon biopsy. It was not possible to determine the anti-inflammatory medications received by all AR patients in this study.

### Isolation and cytokine treatment of Achilles tendon–derived stromal cells

Patient-derived AT or AR tendon stromal cells have been previously described as CD45^neg^CD34^neg^ cells exhibiting fibroblast-like morphology (22). Cells were isolated from patient tissues using previously described protocols (25, 26), and passage 1–2 cells were used for all experiments. Cells were grown until 80% confluence prior to cytokine stimulation. IL-1β is known to induce expression of NF-κB target genes highly expressed in Achilles tendon disease (22). To recapitulate this inflammatory milieu, we therefore investigated the bioactive LM profiles in tendon-derived stromal cells derived from AT and AR patients in the presence of IL-1β (10 ng/ml; MilliporeSigma, Burlington, MA, USA) in medium (DMEM F12 phenol red–free medium; Thermo Fisher Scientific, Waltham, MA, USA) containing 1% heat-inactivated human serum (MilliporeSigma) and 1% penicillin-streptomycin. Nontreated (vehicle only) cells served as experimental controls. After cytokine and vehicle treatment, cells were incubated at 37°C and 5% CO_2_ for 24 h until experimental harvest of the medium and lysate for bioactive LM profiling.

### Modulating bioactive LM profiles of IL-1β–stimulated Achilles tendon–derived stromal cells with 15-epi-LXA_4_ or MaR1

Cells derived from AT or AR patients were seeded at a density of 60,000 cells per well. Once cells were 80% confluent, they were preincubated with 10 nM 15-epi-LXA_4_ (Cayman Chemicals, Ann Arbor, MI, USA) or 10 nM MaR1 (Cayman Chemicals) for 24 h in DMEM F12 phenol red–free medium containing 1% heat-inactivated human serum and 1% penicillin-streptomycin. Cells were stimulated with IL-1β (10 ng/ml) in the presence of medium containing either 15-epi-LXA_4_, MaR1, or vehicle control as previously described (23). Parallel experiments were conducted, and cell lysates were harvested to investigate whether incubating cells in 15-epi-LXA_4_ or MaR1 modulated expression of proinflammatory or proresolving genes expressed by AT and AR cells. The concentration and integrity of mediators used for these incubations were validated using UV-spectrophotometry and liquid chromatography–tandem mass spectrometry in accordance with previously published criteria (27, 28).

### Bioactive LM profiling of Achilles tendon stromal cell incubations

We utilized targeted LM profiling that identifies mediators from all 4 major bioactive metabolomes, including the arachidonic acid metabolome, the eicosapentaenoic acid metabolome, the *n*-3 docosapentaenoic acid (DPA) metabolome, and the docosahexaenoic acid metabolome, simultaneously measuring the substrate, precursors, pathway markers, bioactive mediators, and the further metabolites. Bioactive LM profiling of medium and lysate samples from IL-1β–stimulated AT and AR cells was performed using previously described methodology (29). Calibration curves were obtained for each using authentic compound mixtures and deuterium-labeled LM at 0.78, 1.56, 3.12, 6.25, 12.5, 25, 50, 100, and 200 pg. Linear calibration curves were obtained for each LM, which gave *r*^2^ values of 0.98–0.99.

### Immunocytochemistry for 15-epi-LXA_4_ and MaR1-treated Achilles stromal cells

IL-1β–stimulated AT and AR tendon stromal cells were grown in chamber slides and treated as previously described. After incubation in 15-epi-LXA_4_ or MaR1, cells were fixed in ice-cold methanol for 5 min and washed with PBS. Immunofluorescence staining protocols and image acquisition are adapted from a previously published protocol (23). Tendon stromal cells isolated from AT and AR donors (*n* = 3 each) were incubated with the following primary antibodies: anti-ALX (ab26316; Abcam, Cambridge, MA, USA), anti–arachidonate lipoxygenase (ALOX) 15 (ab119774; Abcam), anti-ALOX12 (ab211506; Abcam), anti–Phospho-STAT-1 (ab29045; Abcam), anti-PDPN (ab10288; Abcam), and anti–IL-6 (ab9324; Abcam) in PBS containing 5% goat serum in saponin for 3 h at room temperature. For negative controls, the primary antibody was substituted for universal isotype control antibodies: cocktail of mouse IgG_1_, IgG_2a_, IgG_2_b, IgG_3_, and IgM (Agilent Technologies, Santa Clara, CA, USA) and rabbit Ig fraction of serum from nonimmunized rabbits, solid phase absorbed. Isotype control staining is shown in Supplemental Fig. S1. Immunofluorescence images were acquired on a Zeiss Laser Scanning Microscope (LSM; Carl Zeiss, Oberkochen, Germany) 710 confocal microscope using a previously described protocol (23).

### Expression of proinflammatory and proresolving genes in AT and AR tendon stromal cells incubated in 15-epi-LXA_4_ or MaR1

AT and AR tendon-derived stromal cells (*n* = 5 each) were seeded at a density of 20,000 cells per well in a 24-well plate. Tendon cells were allowed to reach 80% confluence prior to preincubation with 15-epi-LXA_4_ or MaR1 and subsequent stimulation with IL-1β (10 ng/ml; MilliporeSigma). Nontreated cells (vehicle only, containing 0.1% endotoxin-free bovine serum albumin; MilliporeSigma) served as controls for each experiment. After treatment, cells were then incubated at 37°C and 5% CO_2_ until harvest of the cell lysate for mRNA after 24 h. RNA isolation, cDNA synthesis, and quantitative PCR were performed using previously published protocols (25). Prevalidated Qiagen (Hilden, Germany) primer assays [*ALOX15*, *IL-6*, *STAT-1*, CD90, *IRF5*, *TLR4*, *β-actin*, and glyceraldehyde-3-phosphate dehydrogenase (*GAPDH*)] were used for quantitative PCR. Results were calculated using the ∆∆*C_t_* method using reference genes for human *β-actin* and *GAPDH*. Results were consistent using these reference genes, and data are shown normalized to *β-actin*.

### Phosphokinase signaling in 15-epi-LXA_4_ and MaR1-treated AT tendon stromal cells

A Human Phosphokinase Array Kit (ARY003B; R&D Systems, Minneapolis, MN, USA) was used to investigate the effects of incubating IL-1β–treated AT cells in 15-epi-LXA_4_ or MaR1 on protein phosphokinase signaling pathways. Experimental protocols were performed according to the manufacturer’s instructions on protein lysates harvested after 24 h of incubation in either 15-epi-LXA_4_ or MaR1. Images were captured using a chemiluminescence documentation system (Uvitec, Cambridge, United Kingdom), and densitometry analysis of proteins of interest was performed using ImageJ software (National Institutes of Health, Bethesda, MD, USA).

### Statistical analysis

Statistical analyses were performed using GraphPad Prism 7 (GraphPad Software, La Jolla, CA, USA). Normality was tested using the Shapiro-Wilk normality test. Analysis of bioactive LM profiles from tendon cells derived from patients with AT and AR was performed using multivariate statistical analysis and orthogonal partial least squares discriminant analysis using Statistical Isolinear Multiple Components Analysis (SIMCA) 14.1 software (Sartorius, Göttingen, Germany) following unit variance scaling of LM amounts. Partial least squares discriminant analysis is based on a linear multivariate model that identifies variables that contribute to class separation of observations (cell incubations) on the basis of their variables (LM levels). During LM classification, observations were projected onto their respective class model. The score plot illustrates the systematic clusters among the observations (closer plots presenting higher similarity in the data matrix). Loading plot interpretation identified the variables with the best discriminatory power (variable importance in projection >1) that were associated with tight clusters for LM profiles obtained from incubations with cells from patients with AT or AR. Data are shown as means with sem, where *n* is the biologic replicate (human donor of cells derived from healthy or diseased tendons). Unpaired Student's *t* tests were used to test for differences in LM levels between tendon cells derived from patients with AT and AR. Paired Student's *t* tests were used to test for differences in LM levels after incubating AT or AR cells in the presence of 15-epi-LXA_4_ or MaR1 compared with vehicle control. Pairwise Mann-Whitney *U* tests were used to test for differences between mRNA expression of *ALOX15*, *IL-6*, *STAT-1*, *CD90*, *IRF5*, and *TLR4* in IL-1β–treated tendon stromal cells in the presence or absence of SPMs. Values of *P* < 0.05 were considered statistically significant.

## RESULTS

### Stromal cells derived from patients with AT and AR show distinct LM profiles

**Figure 1:**
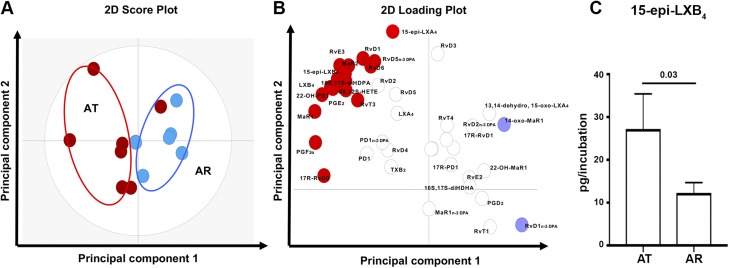
Distinct bioactive LM profiles of stromal cells isolated from patients with AT and AR. Tendon stromal cells (60,000 cells per well) were derived from patients with AT (*n* = 7 donors) or AR (*n* = 7 donors). Cells were cultured in DMEM F12 phenol red–free medium containing 1% heat-inactivated human serum to 80% confluence and incubated for 24 h. Medium and cells were harvested and placed in ice-cold methanol containing deuterium-labeled internal standards. LM were then extracted and profiled. *A*, *B*) Two-dimensional (2D) score plot (*A*) and corresponding 2D loading plot (*B*) of LM-SPM from human tendon–derived stromal cell incubations isolated from AT and AR patients under baseline unstimulated conditions. Gray ellipse in the score plot denotes 95%-confidence regions. 13,14-Dehydro-15-oxo-Lipoxin A4; 22-OH-MaR1, 22-OH-Maresin 1; 22-OH-PD1, 22-OH-Protectin D1; 5S,12S-HETE, 5S,12S–hydroxyeicosatetraenoic acid; 10S,17S-diHDHA, 10S,17S–dihydroxydocosahexaenoic acid; 10S,17S-diHDPA, 10S,17S–dihydroxydocosapentaenoic acid; TXB_2_, thromboxane B2. *C*) Concentrations for mediators found to be differentially regulated between AT and AR tendon stromal cell incubations. Results are shown as means and sem and are representative of *n* = 7 donors per group.

LM profiling of tendon-derived stromal cell cultures from AT and AR patients identified SPMs including D-series resolvins (RvDs) (RvD1, -2, -3, -4, -5, -6, 17R-RvD1, and 17R-RvD3), protectins [protectin D1 (PD1); 17R-PD1; and 10S,17S–dihydroxydocosahexaenoic acid], MaRs (MaR1, MaR2), E-series resolvins (RvEs) (RvE2 and RvE3), T-series resolvins (RvTs) (RvT1 and RvT4), arachidonic acid–derived lipoxins (LXs) (LXA_4_, LXB_4_, 15-epi-LXA_4_, and 15-epi-LXB_4_), and n-3 DPA-derived resolvins (RvD1_n-3 DPA_, -2_n-3 DPA_, and -5_n-3 DPA_), protectins (PD1_n-3 DPA_), and MaRs (MaR1_n-3 DPA_). These mediators were identified in accordance with published criteria that include matching retention times and at least 6 ions in the tandem mass spectrum (27). Cumulative levels of proresolving mediators did not significantly differ between AT- and AR-derived cells. However, multivariate analysis identified differences in the bioactive LM profiles between tendon cells derived from AT and AR patients under baseline unstimulated conditions as demonstrated by the distinct clustering of the LM profiles (**Fig. 1*A, B***). The molecules profiled, together with the concentrations of the individual LMs identified, are listed in Supplemental Table S1. Assessment of individual LM concentrations demonstrated increased levels of 15-epi-LXB_4_ (*P* = 0.03) in AT compared with AR cells (Fig. 1*C*). In incubations of AT cells, we also found increases in inflammation-initiating eicosanoids including prostaglandin (PG) E_2_ and PGF_2α_ (Supplemental Table S1). This increase in both SPMs and eicosanoids in tendon-derived stromal cells from AT patients suggests that, although SPMs are up-regulated in these cells, their concentrations are not sufficient to counterregulate the ongoing inflammatory processes, reminiscent of a dysregulated resolution response characteristic of chronic inflammation.

**Figure 2:**
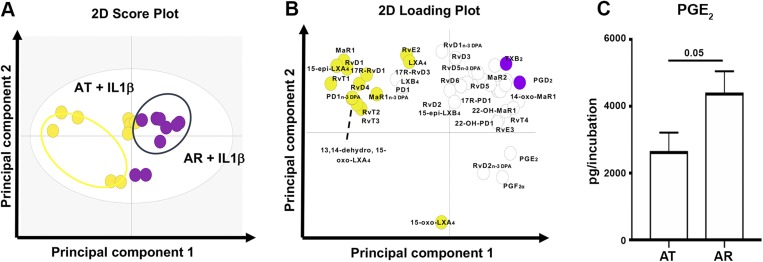
Distinct bioactive LM profiles of IL-1β–stimulated stromal cells isolated from patients with AT and AR. Tendon stromal cells (60,000 cells per well) were derived from patients with AT (*n* = 9 donors) or AR (*n* = 9 donors). Cells were cultured in DMEM F12 phenol red–free medium containing 1% heat-inactivated human serum to 80% confluence and incubated for 24 h in the presence of IL-1β (10 ng/ml). Medium and cells were harvested and placed in ice-cold methanol containing deuterium-labeled internal standards. *A*, *B*) LM were then extracted and profiled. Two-dimensional (2D) score plot (*A*) and corresponding 2D loading plot (*B*) of LM-SPM from human tendon–derived stromal cell incubations isolated from AT and AR patients under IL-1β–stimulated conditions. Gray ellipse in the score plot denotes 95%-confidence regions. 13,14-Dehydro-15-oxo-Lipoxin A4; 22-OH-MaR1, 22-OH-Maresin 1; 22-OH-PD1, 22-OH-Protectin D1; 5S,12S-HETE, 5S,12S–hydroxyeicosatetraenoic acid; 10S,17S-diHDHA, 10S,17S–dihydroxydocosahexaenoic acid; 10S,17S-diHDPA, 10S,17S–dihydroxydocosapentaenoic acid; TXB_2_, thromboxane B2. *C*) Concentrations for mediators found to be differentially regulated between IL-1β–stimulated AT and AR tendon stromal cell incubations. Results are shown as means and sem and are representative of *n* = 9 donors per group.

LM profiling was also performed on AT and AR cells after treatment with IL-1β (10 ng/ml) for 24 h to induce an inflammatory milieu and determine if this cytokine moderated the release of proresolving mediators and inflammation-initiating eicosanoids from these cells. Multivariate analysis for IL-1β–treated AT and AR cells is shown in **Fig. 2*A, B***. The molecules profiled, together with the concentrations of the individual LMs identified, are listed in Supplemental Table S1. In these incubations, IL-1β–treated AR cells showed increased levels of PGE_2_ compared with respective AT cells (*P* = 0.05, Fig. 2*C*). The increased responsiveness of AR cells to cytokine stimulation may reflect the acute inflammatory milieu of these cells.

### 15-Epi-LXA_4_ and MaR1 induce SPM production and regulate inflammation-initiating eicosanoids in Achilles tendon–derived stromal cells

**Figure 3:**
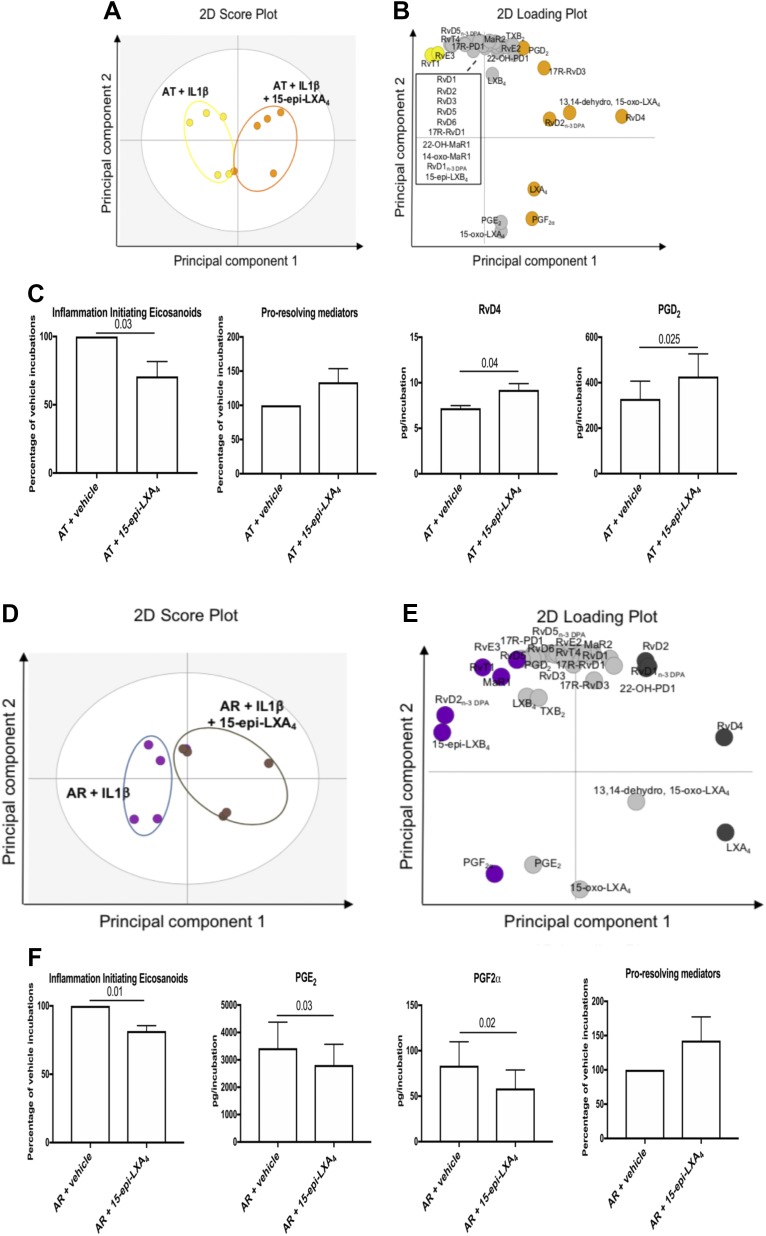
15-Epi-LXA_4_ induces SPMs and dampens inflammation-initiating eicosanoids in IL-1β–stimulated stromal cells isolated from patients with AT and AR. Tendon stromal cells were derived from patients with AT (*n* = 5 donors) or AR (*n* = 5 donors). Cells were incubated with 15-epi-LXA_4_ (10 nM) or vehicle for 24 h at 37°C and then with IL-1β (10 ng/ml) for 24 h. LMs were identified and quantified using LM profiling. *A*, *B*) Two-dimensional (2D) score plot (*A*) and corresponding 2D loading plot (*B*) of LM-SPM from human tendon–derived stromal cell incubations isolated from AT patients under IL-1β–stimulated conditions in the presence of 10 nM 15-epi-LXA_4_ or vehicle only. *C*) Cumulative levels of inflammation-initiating eicosanoids (PGs, thromboxane), proresolving mediators (docosahexaenoic acid-derived RvD, Protectin, MaR, n-3 DPA-derived RvD_n-3 DPA_, PD_n-3 DPA_, MaR1_n-3 DPA_, eicosapentaenoic acid–derived RvE, and arachidonic acid-derived LX), and differentially regulated LMs in IL-1β–stimulated AT tendon stromal cell incubations in the presence of 15-epi-LXA_4_ (10 nM) or vehicle for 24 h. Results are shown as means and sem and are representative of *n* = 5 donors per group. *D*, *E*) The 2D score plot (*D*) and corresponding 2D loading plot (*E*) of plasma LM-SPM from human tendon–derived stromal cell incubations isolated from AR patients under IL-1β–stimulated conditions in the presence of 10 nM 15-epi-LXA_4_ or vehicle only. *F*) Cumulative levels of inflammation-initiating eicosanoids, proresolving mediators, and differentially regulated LMs in IL-1β–stimulated AR tendon stromal cell incubations in the presence of 15-epi-LXA_4_ (10 nM) or vehicle for 24 h. 13,14-Dehydro-15-oxo-Lipoxin A4; 22-OH-MaR1, 22-OH-Maresin 1; 22-OH-PD1, 22-OH-Protectin D1; TXB_2_, thromboxane B2. Results are shown as means and sem and are representative of *n* = 5 donors per group.

In order to gain insight into whether SPMs can regulate tendon stromal cell inflammation, we next tested whether incubation of these cells with 15-epi-LXA_4_ or MaR1, 2 of the SPMs identified in Achilles tendon cell incubations, modulated the bioactive LM profiles of IL-1β–stimulated AT and AR tendon stromal cells. Multivariate analysis identified differences in bioactive LM profiles between IL-1β–stimulated AT cells in the presence of 10 nM 15-epi-LXA_4_ compared with vehicle-only incubations, demonstrated by the distinct clustering of the LM profiles (**Fig. 3*A, B***)_._ The molecules profiled, together with the concentrations of the individual LMs identified, are listed in Supplemental Table S2. Incubation of IL-1β–stimulated AT cells in 15-epi-LXA_4_ significantly regulated inflammation-initiating eicosanoids including PGE_2_, PGF_2α_, and thromboxane B2 (Fig. 3*C*, *P* = 0.03). We also found that incubating AT cells in 15-epi-LXA_4_ up-regulated production of proresolving mediators and significantly increased RvD4 (*P* = 0.04) and PGD_2_ (*P* = 0.025) compared with respective vehicle controls (Fig. 3*C* and Supplemental Table S2)_._ Assessment of LM profiles from IL-1β–stimulated AR cells incubated with 10 nM 15-epi-LXA_4_ compared with vehicle-only incubations demonstrated distinct clustering of the LM profiles (Fig. 3*D, E*). The molecules profiled, together with the concentrations of the individual LMs identified, are listed in Supplemental Table S2. Incubation of IL-1β–stimulated AR cells in 15-epi-LXA_4_ regulated all inflammation-initiating eicosanoids (*P* = 0.01), specifically decreasing PGE_2_ (*P* = 0.03) and PGF_2α_ (*P* = 0.02) (Fig. 3*F* and Supplemental Table S2). We also found that incubating AR cells in 15-epi-LXA_4_ induced production of all proresolving mediators compared with respective vehicle controls (Fig. 3*F*).

**Figure 4:**
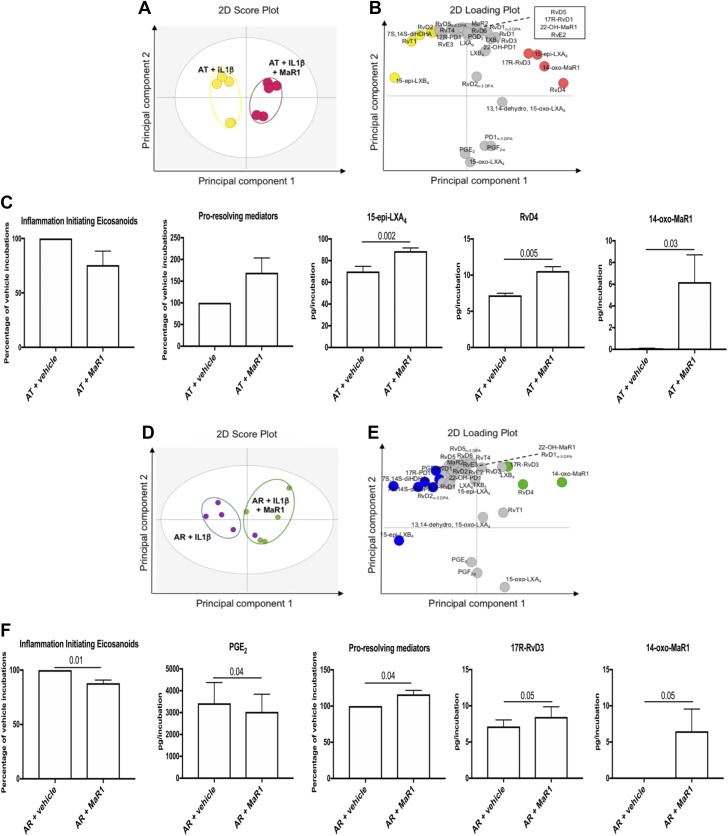
MaR1 induces SPM and dampens inflammation-initiating eicosanoids in IL-1β–stimulated stromal cells isolated from patients with AT and AR. Tendon stromal cells were derived from patients with AT (*n* = 5 donors) or AR (*n* = 5 donors). Cells were incubated with MaR1 (10 nM) or vehicle for 24 h at 37°C and then with IL-1β (10 ng/ml) for 24 h. LMs were identified and quantified using LM profiling. *A*, *B*) Two-dimensional (2D) score plot (*A*) and corresponding 2D loading plot (*B*) of LM-SPM from human tendon–derived stromal cell incubations isolated from AT patients under IL-1β–stimulated conditions in the presence of 10 nM MaR1 or vehicle only. *C*) Cumulative levels of inflammation-initiating eicosanoids, proresolving mediators, and differentially regulated proresolving mediators in IL-1β–stimulated AT tendon stromal cell incubations in the presence of MaR1 (10 nM) or vehicle for 24 h. Results are shown as means and sem and are representative of *n* = 5 donors per group. *D*, *E*) A 2D score plot (*D*) and corresponding 2D loading plot (*E*) of plasma LM-SPM from human tendon–derived stromal cell incubations isolated from AR patients under IL-1β–stimulated conditions in the presence of 10 nM MaR1 or vehicle only. *F*) Cumulative levels of inflammation-initiating eicosanoids, proresolving mediators, and differentially regulated LMs in IL-1β–stimulated AR tendon stromal cell incubations in the presence of MaR1(10 nM) or vehicle for 24 h. 13,14-Dehydro-15-oxo-Lipoxin A4; 22-OH-MaR1, 22-OH-Maresin 1; 22-OH-PD1, 22-OH-Protectin D1; TXB_2_, thromboxane B2. Results are shown as means and sem and are representative of *n* = 5 donors per group.

We next investigated whether incubation of AT cells with MaR1 altered LM profiles in response to IL-1β stimulation. Multivariate analysis demonstrated that MaR1 incubation led to a shift in the bioactive LM profiles of IL-1β–stimulated AT cells compared with vehicle-only incubations (**Fig. 4*A, B***). The molecules profiled, together with the concentrations of the individual LMs identified, are listed in Supplemental Table S2. Incubation of IL-1β–stimulated AT cells with MaR1 dampened inflammation-initiating eicosanoids and induced release of proresolving mediators (Fig. 4*C*). This treatment increased specific SPMs including 15-epi-LXA_4_ (*P* = 0.002), RvD4 (*P* = 0.005), and the MaR metabolite 14-oxo-MaR1 (*P* = 0.03) compared with respective vehicle controls (Fig. 4*C* and Supplemental Table S2).

MaR1 incubation also resulted in a shift in the LM profile of AR cells, as demonstrated by a separation in the LM clusters obtained in orthogonal partial least squares discriminant analysis (Fig. 4*D, E*). The molecules profiled, together with the concentrations of the individual LMs identified, are listed in Supplemental Table S2. Incubation of IL-1β–stimulated AR cells with MaR1 regulated all inflammation-initiating eicosanoids (*P* = 0.01), specifically decreasing PGE_2_ (*P* = 0.04) (Fig. 4*F*). Incubating AR cells in MaR1 also increased the production of all proresolving mediators (*P* = 0.04). This treatment increased specific SPMs including 17R-RvD3 (*P* = 0.05) and the MaR metabolite 14-oxo-MaR1 (*P* = 0.05) compared with respective vehicle controls (Fig. 4*F*). Together, these findings demonstrate that 15-epi-LXA_4_ and MaR1 counterregulate IL-1β–initiated inflammation in tendon stromal cells derived from patients with AT and AR.

### 15-Epi-LXA_4_ and MaR1 up-regulate the expression of SPM biosynthetic enzymes and proresolving receptor ALX in Achilles tendon stromal cells

**Figure 5:**
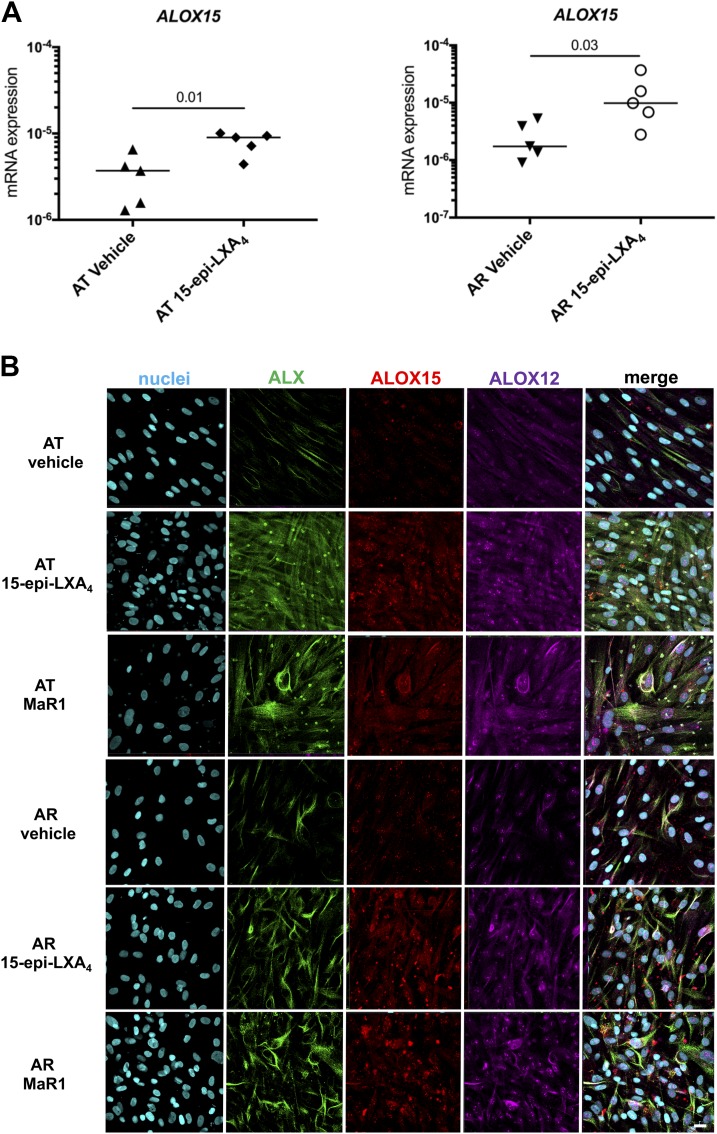
15-Epi-LXA_4_ and MaR1 induce SPM synthetic enzymes and ALX in Achilles tendon stromal cells. Tendon stromal cells were derived from patients with AT (*n* = 5 donors) or AR (*n* = 5 donors). Cells were incubated with 15-epi-LXA_4_ (10 nM) or vehicle for 24 h at 37°C and then with IL-1β (10 ng/ml) for 24 h. *A*) Incubation in 15-epi-LXA_4_ significantly induced *ALOX15* mRNA in both AT (*P* = 0.01) and AR (*P* = 0.03) cells compared with respective vehicle controls. Gene expression is normalized to β-actin; bars show median values. *B*) Representative images of immunocytochemistry for the proresolving receptor ALX (green) and SPM synthetic enzymes ALOX12 (violet) and ALOX15 (red) in IL-1β–stimulated AT and AR tendon stromal cells incubated in 10 nM 15-epi-LXA_4,_ 10 nM MaR1, or vehicle control for 24 h. Cyan represents Popo-1 nuclear counterstain. All images are representative of *n* = 3 donors. Scale bar, 20 μm.

We next investigated the mechanisms by which 15-epi-LXA_4_ and MaR1 potentiated release of proresolving mediators. Incubation of IL-1β–stimulated AT or AR cells in 15-epi-LXA_4_ induced expression of *ALOX15* mRNA relative to IL-1β–stimulated vehicle controls (*P* = 0.01 and *P* = 0.03, respectively; **Fig. 5*A***). Incubation of IL-1β–stimulated AT or AR cells in 15-epi-LXA_4_ or MaR1 induced ALOX12 and ALOX15 when compared with respective vehicle controls (Fig. 5*B*). We next investigated whether these SPMs also up-regulated the expression of the proresolving receptor ALX, given its role in the termination of musculoskeletal inflammation (30, 31). Indeed both 15-epi-LXA_4_ and MaR1 up-regulated the expression of this receptor in both AT and AR cells, with a marked increase observed in AT cells.

### 15-Epi-LXA_4_ and MaR1 moderate the inflammatory phenotype of Achilles tendon stromal cells, dampening proinflammatory signaling pathways

**Figure 6:**
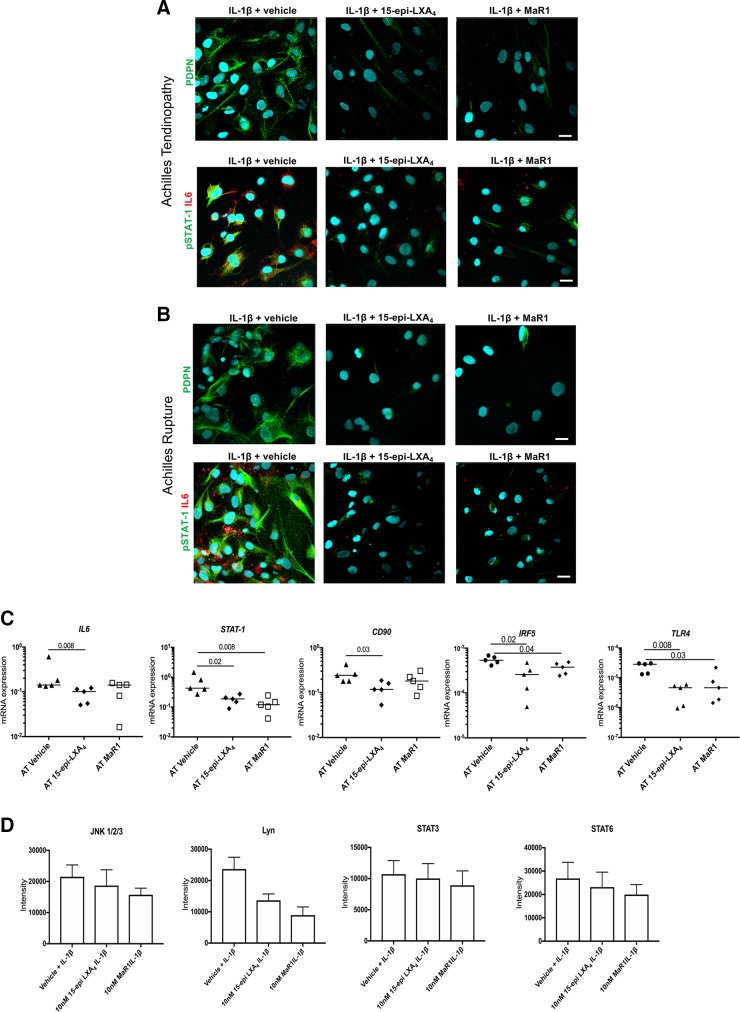
15-Epi-LXA_4_ and MaR1 moderate the proinflammatory phenotype of Achilles tendon stromal cells. Tendon stromal cells were derived from patients with AT or AR. *A*, *B*) Representative images of immunocytochemistry for established markers of tendon inflammation including PDPN (green), phosphorylated STAT-1 (green), and IL-6 (red) in IL-1β–stimulated AT (*A*) and AR (*B*) tendon stromal cells incubated in 10 nM 15-epi-LXA_4_, 10 nM MaR1, or vehicle control for 24 h. Cyan represents Popo-1 nuclear counterstain. All images are representative of *n* = 3 donors. Scale bars, 20 μm. *C*) mRNA expression of markers of tendon inflammation including *IL-6*, *STAT-1*, pathogenic fibroblast marker *CD90*, *IRF5,* and *TLR4* in IL-1β–stimulated AT cells (*n* = 5 donors) incubated in either 10 nM 15-epi-LXA_4_, 10 nM MaR1, or vehicle control for 24 h. Gene expression is normalized to β-actin, and bars show median values. *D*) Densitometric analysis was acquired using ImageJ software to identify the effects of incubating IL-1β–treated AT cells in 15-epi-LXA_4_ or MaR1 on protein phosphokinase signaling pathways JNK1/2/3, Lyn, STAT3, and STAT6. Results are shown as means and sem and are representative of *n* = 3 donors per group relative to respective vehicle control–treated cells.

We next assessed whether 15-epi-LXA_4_ and MaR1 also regulated known markers of tendon inflammation in patient-derived AT and AR cells. Incubation of IL-1β–stimulated AT and AR cells in 15-epi-LXA_4_ or MaR1 reduced fibroblast activation marker PDPN, phosphorylated STAT-1, and IL-6 after 24 h of incubation compared with respective vehicle controls (**Fig. 6*A, B***). In IL-1β–stimulated AT cells, 15-epi-LXA_4_ reduced *IL-6*, *STAT-1*, *CD90*, *IRF5*, and *TLR4* mRNA expression compared with vehicle control–treated cells (*P* = 0.008, *P* = 0.02, *P* = 0.03, *P* = 0.02, and *P* = 0.008, respectively; Fig. 6*C*). Incubating IL-1β–stimulated AT cells in MaR1 reduced *STAT-1*, *IRF5*, and *TLR4* mRNA compared with vehicle controls (*P* = 0.008, *P* = 0.04, and *P* = 0.03, respectively; Fig. 6*C*). In these incubations, 15-epi-LXA_4_ or MaR1 regulated phosphokinases including c-Jun N-terminal kinase (JNK)1/2/3 (phosphorylation sites T183/Y185, T221/Y223), Lyn (Y397), STAT-3 (Y705), and STAT-6 (Y641) compared with respective vehicle control–treated cells (Fig. 6*D*).

## DISCUSSION

Tissue-resident stromal cells including fibroblasts are emerging as important cell types sustaining chronic inflammation in common soft tissue diseases of the joint (32). Tendon fibroblasts are implicated in the development of chronic inflammation through stromal fibroblast activation and inflammation memory (22, 26). We previously identified bioactive LM profiles in tendon stromal cells derived from patients with shoulder tendon tears, identifying dysregulated resolution responses in cells isolated from diseased compared with healthy donors (23). In the current study, we investigated whether tendon stromal cells derived from AT and AR patients showed distinct bioactive LM profiles. AT and AR both represent clinical manifestations of established tendon disease. Although AR patients may have established tendon pathology prior to rupture, the episode of recent traumatic injury associated with the event of tendon rupture presents a superimposed phase of acute inflammation on an already chronically inflamed tendon (22). Using tendon stromal cells isolated from fresh patient tissues, we identified differences in bioactive LM profiles between AT and AR tendon stromal cells under baseline unstimulated conditions. AT cells showed significantly increased levels of 15-epi-LXB_4_ compared with AR cells. AT cells also showed increased levels of inflammation-initiating eicosanoids PGE_2_ and PGF_2α_ compared with AR cells. These findings suggest that, although SPMs are up-regulated in AT-derived stromal cells, their concentrations are not sufficient to counterregulate the ongoing inflammatory process, which is reminiscent of the dysregulated resolution responses we previously identified in shoulder tendon tears (23). We subsequently stimulated AT and AR cells with IL-1β because it is known to induce expression of NF-κB target genes highly expressed in human tendon disease (22, 25), therefore recapitulating an inflammatory milieu. In these incubations, we found increased PGE_2_ levels in AR compared with respective AT cells. This increased responsiveness of AR cells may be attributable to the acute inflammatory milieu associated with traumatic AR, whereby cells are “primed” as a consequence of a recent episode of acute inflammation. Therefore, the differences in the bioactive LM profiles between AT and AR cells may reflect the temporal effects of disease stage associated with different clinical manifestations of Achilles tendon disease. Previous studies have identified temporal release of SPMs during the resolution phase of inflammatory responses. A study utilizing self-limiting exudates identified a distinct time frame of the proresolving mediator RvD3, which appeared late in the resolution phase (33). MaR1 was also shown to be temporally regulated in *Escherichia coli* leukocyte infectious exudates (34). In the current study of patient tissue samples, it was not possible to obtain sequential tendon biopsies, prohibiting the study of temporal metabolipidomics within individual patients.

Having identified that a select group of proresolving mediators were released by patient-derived Achilles tendon cells, we next investigated the biologic actions of these autacoids, and their capacity to regulate molecular aspects of inflammation in AT and AR. Incubating IL-1β–stimulated AT or AR cells in 15-epi-LXA_4_ or MaR1 regulated inflammation-initiating eicosanoids and up-regulated concentrations of SPMs in these incubations. We previously identified these SPM-induced analogous responses in tendon stromal cells isolated from patients with shoulder tendon tears, and we showed that the effects of these SPMs were blunted in patient compared with healthy volunteer tendon-derived stromal cells (23). We also identified the MaR1 metabolite 14-oxo-MaR1 in MaR1-incubated cells isolated from shoulder tendon tear patients (23). The current study confirmed the presence of this metabolite in MaR1-incubated AT and AR cells, suggesting that common shared mechanisms exist for MaR1 metabolism in stromal fibroblasts isolated from positional shoulder and energy-storing Achilles tendons. In the current study, incubation in 15-epi-LXA_4_ specifically up-regulated RvD4 and PGD_2_ in IL-1β–stimulated AT cells. Conversely, incubation with MaR1 induced 15-epi-LXA_4_ and RvD4 in AT cells and 17-R-RvD3 in AR cells. Although 15-epi-LXA_4_ and MaR1 up-regulated concentrations of specific SPMs in these incubations, both treatments reduced known markers of tendon inflammation, including IL-6, STAT-1, and fibroblast activation markers CD90 and PDPN, which are associated with disease severity (22, 25, 26). Collectively, these findings suggest that incubation of IL-1β–stimulated AT and AR tendon-derived stromal cells in 15-epi-LXA_4_ or MaR1 regulate inflammation-initiating eicosanoids, potentiate the release of proresolving mediators, and moderate the proinflammatory phenotype of these patient-derived cells.

We next investigated the mechanism of action underpinning these observations. Incubation of AT and AR cells in either 15-epi-LXA_4_ or MaR1 induced SPM synthetic enzymes ALOX12 and ALOX15 and the proresolving receptor ALX. 15-Epi-LXA_4_ also profoundly induced ALX in IL-1β–stimulated AT cells. We also identified incubation of IL-1β–stimulated AT cells in 15-epi-LXA_4_– or MaR1-regulated phosphokinases including JNK1/2/3, Lyn, STAT-3, and STAT-6. Binding of 15-epi-LXA_4_ to ALX is known to suppress kinases downstream of MAPK, including JNK (35). The findings from our study suggest that 15-epi-LXA_4_ or MaR1 regulate IL-6 *via* suppressing STAT-3 signaling in AT stromal cells. Suppression of STAT-6 signaling may modulate IL-4 and IL-13 responsive genes known to drive fibrosis in the advanced stages of tendon disease (25, 36). Lyn is an Src family kinase regulating multiple aspects of immune signaling (37, 38). Ban *et al.* (39) recently identified Lyn as a specific suppressor of the TLR–myeloid differentiation primary response 88–IRF5 pathway, highlighting the importance of this kinase in regulating IRF5 activity in immune homeostasis in Systemic Lupus Erythematosus. We previously identified IRF5 and TLR4 as being highly expressed in tissues from patients with AT and AR (22). In the current study, incubation of IL-1β–stimulated AT cells in 15-epi-LXA_4_ or MaR1 suppressed *IRF5* and *TLR4* mRNA expression compared with vehicle control–treated cells. Therefore, 15-epi-LXA_4_– or MaR1-induced regulation of Lyn presents a potential means of regulating proinflammatory molecules including IRF5 and TLR4 in Achilles tendon disease.

It was not possible to determine the anti-inflammatory medications received by AR patients in this study. Of note, in the event that these patients had taken NSAIDs, it is unlikely that these will have a significant impact on the biosynthesis of LMs by cells in culture because these therapeutics are anticipated to be diluted out in culture. In addition, for those anti-inflammatories that irreversibly bind to and inhibit their target enzymes, such as aspirin, the target protein is likely turned over in culture and, therefore, the activity of these drugs is lost.

In summary, we identified some differences in bioactive LM profiles between tendon stromal cells isolated from patients with AT and AR, which likely reflect the temporal effects of disease stage. We demonstrate that 15-epi-LXA_4_ and MaR1 regulate expression of proinflammatory molecules including PGE_2_, PDPN, CD90, STAT-1, IL-6, IRF5, and TLR4 and dampen phosphokinases including JNK1/2/3, Lyn, STAT-3, and STAT-6. These SPMs also up-regulated proresolving mediators in IL-1β–stimulated AT and AR cells. Collectively, these findings suggest that 15-epi-LXA_4_ and MaR1 are potential future therapies to counterregulate inflammatory processes in cells isolated from patients with AT and AR.

## Supplementary Material

This article includes supplemental data. Please visit *http://www.fasebj.org* to obtain this information.

## Abbreviations

15-epi-LXA_4_: 15-epi-Lipoxin A_4_
ALOX: arachidonate lipoxygenase
AR: Achilles rupture
AT: Achilles tendinopathy
DPA: docosapentaenoic acid
HVI: high-volume injection
IRF: IFN regulatory factor
JNK: c-Jun N-terminal kinase
LM: lipid mediator
LX: lipoxin
MaR: maresin
NSAID: nonsteroidal anti-inflammatory drug
PD1: protectin D1
PDPN: podoplanin
PG: prostaglandin
RvD: D-series resolvin
RvE: E-series resolvin
RvT: T-series resolvin
SPM: specialized proresolving mediator
STAT: signal transducer and activator of transcription
