# Supplementary material for: 15-Epi-LXA4 and MaR1 counter inflammation in stromal cells from patients with Achilles tendinopathy and rupture
Source: FASEB J. 2019 Mar 27;33(7):8043–54. doi: 10.1096/fj.201900196R (PMC6593888; doi:10.1096/fj.201900196R)
Supplement: Supplementary file 1 [file fj.201900196R.sd1.docx]

**Figure S1. Isotype control staining of Achilles tendon derived stromal cells.** Representative confocal immunofluorescence images showing merged images of stromal cells isolated from patients with Achilles tendinopathy, stained with isotype control antibodies for mouse IgG_1_, IgG_2a_, IgG_2b_ and rabbit IgG fractions. Cyan represents POPO-1 nuclear counterstain. Scale bar, 20μm.
