# Supplementary figures and images for: 15-Epi-LXA4 and MaR1 counter inflammation in stromal cells from patients with Achilles tendinopathy and rupture
Source: FASEB J. 2019 Mar 27;33(7):8043–54. doi: 10.1096/fj.201900196R (PMC6593888; doi:10.1096/fj.201900196R)

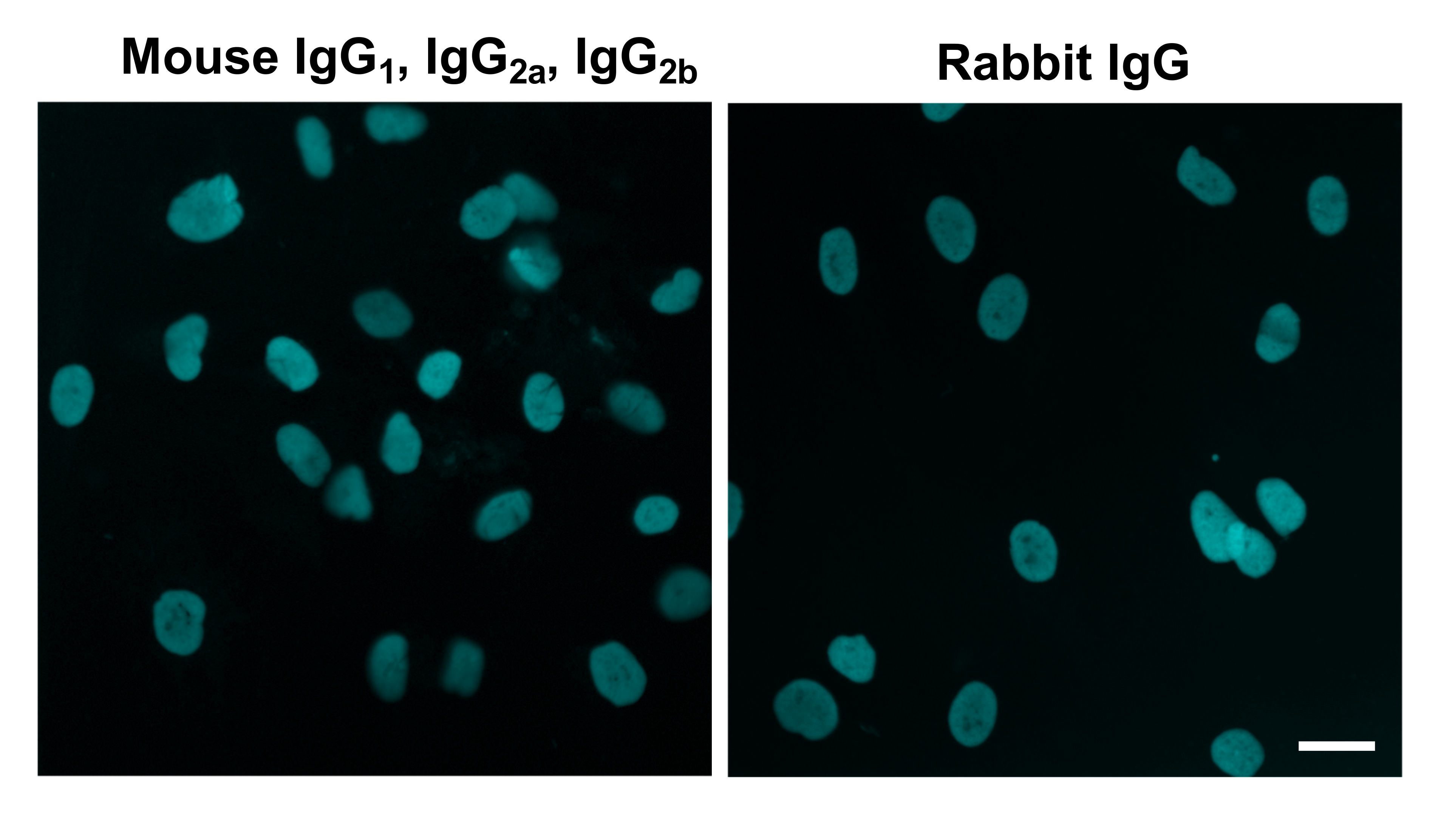

Supplement: Supplementary file 2 [file fj.201900196R.sf1.tif]
