# Supplementary material for: 15-Epi-LXA4 and MaR1 counter inflammation in stromal cells from patients with Achilles tendinopathy and rupture
Source: FASEB J. 2019 Mar 27;33(7):8043–54. doi: 10.1096/fj.201900196R (PMC6593888; doi:10.1096/fj.201900196R)
Supplement: Supplementary file 3 [file fj.201900196R.st1.docx]

**Supplemental Table 1. LM-SPM profiles of tendon stromal cells derived from patients with Achilles tendinopathy and rupture under baseline and IL-1β stimulated conditions.**

| **Tendon stromal cells lipid mediator levels**  **pg/incubation** | **AT** | | | **AR** | | | **AT + IL1β** | | | **AR + IL1β** | | |
| --- | --- | --- | --- | --- | --- | --- | --- | --- | --- | --- | --- | --- |
| **DHA bioactive metabolome** | **Mean** | **±** | **SEM** | **Mean** | **±** | **SEM** | **Mean** | **±** | **SEM** | **Mean** | **±** | **SEM** |
| RvD1 | 9.12 | ± | 1.79 | 6.83 | ± | 0.30 | 6.69 | ± | 0.92 | 5.27 | ± | 0.36 |
| RvD2 | 6.29 | ± | 0.96 | 4.78 | ± | 0.98 | 3.98 | ± | 0.80 | 2.96 | ± | 0.65 |
| RvD3 | 10.41 | ± | 1.06 | 10.40 | ± | 1.11 | 8.97 | ± | 1.31 | 8.71 | ± | 1.53 |
| RvD4 | 17.66 | ± | 2.29 | 16.19 | ± | 2.26 | 16.88 | ± | 4.14 | 12.75 | ± | 2.62 |
| RvD5 | 83.75 | ± | 14.67 | 69.90 | ± | 16.60 | 67.88 | ± | 14.07 | 69.93 | ± | 17.36 |
| RvD6 | 36.31 | ± | 3.01 | 31.10 | ± | 2.80 | 21.05 | ± | 3.78 | 21.06 | ± | 3.42 |
| 17R-RvD1 | 3.75 | ± | 1.37 | 4.05 | ± | 0.77 | 3.41 | ± | 0.41 | 2.44 |  | 0.45 |
| 17R-RvD3 | 14.45 | ± | 2.23 | 11.46 | ± | 1.31 | 10.31 | ± | 1.77 | 10.36 |  | 1.62 |
| PD1 | 6.15 | ± | 2.92 | 3.70 | ± | 1.58 | 1.98 | ± | 1.08 | 1.56 | ± | 0.68 |
| 10S,17SdiHDHA | 218.67 | ± | 76.90 | 224.66 | ± | 69.13 | 178.43 | ± | 48.98 | 170.08 | ± | 54.20 |
| 22OHPD1 | 15.37 | ± | 4.96 | 5.95 | ± | 2.98 | 6.32 | ± | 2.84 | 5.17 | ± | 2.03 |
| 17R-PD1 | 5.76 | ± | 2.34 | 5.79 | ± | 1.75 | 5.80 | ± | 2.06 | 5.50 | ± | 2.12 |
| Maresin1 | 54.30 | ± | 27.64 | 17.87 | ± | 3.08 | 19.13 | ± | 4.70 | 10.19 | ± | 2.70 |
| Maresin2 | 15.82 | ± | 2.58 | 10.51 | ± | 2.36 | 8.88 | ± | 1.73 | 8.77 | ± | 2.11 |
| 22-OH-MaR1 | 21.49 | ± | 7.59 | 22.98 | ± | 5.49 | 21.30 | ± | 4.02 | 18.74 | ± | 4.50 |
| 14-oxo-MaR1 | 0.11 | ± | 0.08 | 0.22 | ± | 0.09 | 0.15 | ± | 0.13 | 0.24 | ± | 0.13 |
| 7S,14S diHDHA | 128.86 | ± | 18.26 | 90.89 | ± | 14.74 | 75.47 | ± | 17.38 | 71.40 | ± | 18.27 |
| 4,14-diHDHA | 79.15 | ± | 19.42 | 75.85 | ± | 17.35 | 33.50 | ± | 6.32 | 32.70 | ± | 9.57 |
| **n-3 DPA bioactive metabolome** | | | | | | | | | | | | |
| RvT1 | 4.26 | ± | 1.00 | 7.44 | ± | 3.79 | 6.74 | ± | 2.37 | 3.28 | ± | 1.05 |
| RvT2 | 0.08 | ± | 0.08 | 0.00 | ± | 0.00 | 0.14 | ± | 0.10 | 0.08 | ± | 0.09 |
| RvT3 | 0.14 | ± | 0.10 | 0.06 | ± | 0.07 | 0.21 | ± | 0.20 | 0.04 | ± | 0.04 |
| RvT4 | 18.13 | ± | 7.69 | 16.70 | ± | 7.80 | 14.26 | ± | 5.72 | 14.40 | ± | 5.66 |
| RvD1_n3 DPA_ | 11.80 | ± | 3.56 | 13.57 | ± | 1.34 | 12.78 | ± | 1.66 | 12.17 | ± | 1.61 |
| RvD2_n3 DPA_ | 2.56 | ± | 1.02 | 2.49 | ± | 0.87 | 2.62 | ± | 0.62 | 2.41 | ± | 0.57 |
| RvD5_n3 DPA_ | 56.32 | ± | 7.26 | 44.37 | ± | 8.31 | 43.92 | ± | 7.63 | 41.44 | ± | 8.92 |
| PD1n_3 DPA_ | 15.54 | ± | 7.80 | 7.04 | ± | 5.29 | 8.82 | ± | 4.33 | 4.74 | ± | 3.33 |
| 10S,17S-diHDPA | 53.28 | ± | 6.01 | 40.21 | ± | 6.25 | 31.50 | ± | 5.97 | 33.21 | ± | 8.25 |
| MaR1n3 DPA | 1.96 | ± | 0.88 | 1.97 | ± | 0.85 | 2.40 | ± | 1.08 | 1.88 | ± | 0.88 |
| 7S,14S-diHDPA | 111.31 | ± | 29.54 | 60.61 | ± | 26.71 | 57.00 | ± | 25.07 | 62.11 | ± | 24.49 |
| **EPA bioactive metabolome** | | | | | | | | | | | | |
| RvE1 | 0.00 | ± | 0.00 | 0.00 | ± | 0.00 | 0.00 | ± | 0.00 | 0.00 | ± | 0.00 |
| RvE2 | 76.89 | ± | 15.96 | 81.36 | ± | 9.50 | 81.29 | ± | 10.52 | 71.83 | ± | 11.81 |
| RvE3 | 6.81 | ± | 2.27 | 3.65 | ± | 1.71 | 4.11 | ± | 1.42 | 3.62 | ± | 1.51 |
| **AA bioactive metabolome** | | | | | | | | | | | | |
| LXA_4_ | 9.62 | ± | 2.21 | 8.16 | ± | 1.43 | 6.57 | ± | 1.11 | 5.98 | ± | 1.04 |
| LXB_4_ | 70.29 | ± | 45.95 | 14.27 | ± | 3.72 | 14.35 | ± | 6.23 | 12.18 | ± | 5.44 |
| 5,15-diHETE | 3265.47 | ± | 951.06 | 3115.97 | ± | 860.27 | 2357.10 | ± | 641.81 | 2481.35 | ± | 756.95 |
| 15-epi-LXA_4_ | 104.03 | ± | 13.84 | 95.70 | ± | 6.78 | 94.98 | ± | 11.11 | 77.77 | ± | 5.37 |
| 15-epi-LXB_4_ | 27.22 | ± | 8.87* | 12.28 | ± | 2.60 | 12.90 | ± | 2.00 | 11.65 | ± | 2.40 |
| 13,14-dihydro-15-oxo-LXA_4_ | 19.08 | ± | 1.17 | 19.89 | ± | 0.81 | 20.78 | ± | 2.51 | 18.23 | ± | 1.39 |
| 15-oxo-LXA_4_ | 0.00 | ± | 0.00 | 0.00 | ± | 0.00 | 7.75 | ± | 5.47 | 8.62 | ± | 6.07 |
| LTB_4_ | 0.00 | ± | 0.00 | 0.00 | ± | 0.00 | 0.00 | ± | 0.00 | 0.00 | ± | 0.00 |
| 5,12 diHETE | 744.25 | ± | 115.54 | 518.65 | ± | 111.30 | 405.54 | ± | 89.83 | 393.17 | ± | 101.37 |
| 6-trans-LTB_4_ | 287.54 | ± | 70.11 | 284.02 | ± | 67.99 | 197.11 | ± | 41.94 | 212.65 | ± | 51.73 |
| 12-epi-6-trans-LTB_4_ | 286.20 | ± | 80.54 | 268.77 | ± | 76.01 | 186.03 | ± | 45.63 | 187.37 | ± | 50.00 |
| PGD_2_ | 241.91 | ± | 82.99 | 288.43 | ± | 50.19 | 298.00 | ± | 46.70 | 351.76 | ± | 57.16 |
| PGE_2_ | 155.33 | ± | 54.35 | 76.23 | ± | 3.35 | 2801.92 | ± | 534.07 | 4047.91 | ± | 707.58* |
| PGF_2α_ | 69.95 | ± | 36.81 | 17.11 | ± | 5.47 | 73.07 | ± | 15.00 | 91.13 | ± | 19.29 |
| TXB_2_ | 21.07 | ± | 2.11 | 19.81 | ± | 2.11 | 15.58 | ± | 2.10 | 18.37 | ± | 3.10 |

Tendon stromal cells (60,000 cells per well) were derived from patients with Achilles tendinopathy (AT) or Achilles rupture (AR) and incubated under baseline unstimulated conditions (n=7 donors each) or in the presence of 10ngml^-1^ IL1β for 24 hrs (n=9 donors each). Cell incubations were terminated using ice-cold methanol containing internal standards and lipid mediators (LM) were identified and quantified using LM-profiling (see methods for details). Results are expressed as pg/incubation. Data are shown as mean ± SEM. * p ≤ 0.05 comparison between respective AT and AR tendon stromal cells. The detection limit was ~ 0.1 pg. 0.0, Below limits of detection.
