# Supplementary material for: 15-Epi-LXA4 and MaR1 counter inflammation in stromal cells from patients with Achilles tendinopathy and rupture
Source: FASEB J. 2019 Mar 27;33(7):8043–54. doi: 10.1096/fj.201900196R (PMC6593888; doi:10.1096/fj.201900196R)
Supplement: Supplementary file 4 [file fj.201900196R.st2.docx]

**Supplemental Table 2. LM-SPM profiles of IL-1β stimulated tendon stromal cells derived from patients with Achilles tendinopathy and rupture in the presence of 15-epi-LXA_4_ or MaR1.**

| **Tendon stromal cells lipid mediator levels**  **pg/incubation** | **AT + IL1β** | | | **AT+IL1β+15-epi-LXA_4_** | | | **AT+IL1β+MaR1** | | | **AR + IL1β** | | | **AR+IL1β+15-epi-LXA_4_** | | | **AR+IL1β+MaR1** | | |
| --- | --- | --- | --- | --- | --- | --- | --- | --- | --- | --- | --- | --- | --- | --- | --- | --- | --- | --- |
| **DHA bioactive metabolome** | **Mean** | **±** | **SEM** | **Mean** | **±** | **SEM** | **Mean** | **±** | **SEM** | **Mean** | **±** | **SEM** | **Mean** | **±** | **SEM** | **Mean** | **±** | **SEM** |
| RvD1 | 5.12 | ± | 0.81 | 5.29 | ± | 0.70 | 5.79 | ± | 0.73 | 5.19 | ± | 0.53 | 5.53 | ± | 0.74 | 4.91 | ± | 0.66 |
| RvD2 | 4.84 | ± | 1.18 | 4.08 | ± | 1.07 | 3.67 | ± | 0.76 | 3.68 | ± | 0.96 | 3.97 | ± | 0.70 | 4.06 | ± | 0.97 |
| RvD3 | 6.97 | ± | 1.53 | 6.55 | ± | 1.59 | 7.97 | ± | 2.06 | 5.82 | ± | 1.24 | 5.60 | ± | 0.92 | 6.84 | ± | 1.36 |
| RvD4 | 7.21 | ± | 0.28 | 9.21 | ± | 0.77* | 10.57 | ± | 0.67* | 6.81 | ± | 0.52 | 7.38 | ± | 0.65 | 8.04 | ± | 1.21 |
| RvD5 | 83.21 | ± | 22.33 | 88.60 | ± | 24.76 | 88.70 | ± | 26.06 | 96.52 | ± | 22.96 | 95.42 | ± | 26.59 | 90.44 | ± | 24.39 |
| RvD6 | 21.54 | ± | 5.92 | 19.85 | ± | 5.10 | 22.72 | ± | 6.64 | 24.40 | ± | 4.65 | 21.43 | ± | 4.50 | 25.26 | ± | 6.08 |
| 17R-RvD1 | 3.09 | ± | 0.58 | 3.15 | ± | 0.58 | 3.04 | ± | 0.84 | 2.65 |  | 0.58 | 2.76 | ± | 0.50 | 2.40 | ± | 0.56 |
| 17R-RvD3 | 7.16 | ± | 0.59 | 7.97 | ± | 1.56 | 9.03 | ± | 1.40 | 7.16 |  | 0.90 | 6.79 | ± | 0.74 | 8.46 | ± | 1.57* |
| PD1 | 0.00 | ± | 0.00 | 0.00 | ± | 0.00 | 0.00 | ± | 0.00 | 0.00 | ± | 0.00 | 0.00 | ± | 0.00 | 0.00 | ± | 0.00 |
| 10S,17SdiHDHA | 210.38 | ± | 78.50 | 179.38 | ± | 74.94 | 199.57 | ± | 81.38 | 244.60 | ± | 72.96 | 256.87 | ± | 80.61 | 257.06 | ± | 81.68 |
| 22OHPD1 | 11.15 | ± | 3.57 | 13.01 | ± | 4.96 | 13.39 | ± | 5.07 | 9.11 | ± | 2.11 | 9.59 | ± | 2.67 | 9.69 | ± | 2.68 |
| 17R-PD1 | 8.05 | ± | 3.25 | 6.40 | ± | 2.68 | 5.18 | ± | 2.20 | 8.24 | ± | 3.16 | 7.59 | ± | 3.64 | 6.51 | ± | 3.01 |
| Maresin1 | 11.48 | ± | 4.85 | 9.33 | ± | 4.16 | 584.51 | ± | 70.91 | 8.17 | ± | 3.46 | 7.40 | ± | 4.28 | 794.95 | ± | 129.13 |
| Maresin2 | 10.66 | ± | 2.73 | 11.46 | ± | 3.01 | 11.44 | ± | 3.26 | 11.90 | ± | 2.91 | 12.65 | ± | 3.64 | 11.97 | ± | 3.09 |
| 22-OH-MaR1 | 24.35 | ± | 6.72 | 23.47 | ± | 7.17 | 25.78 | ± | 6.13 | 24.92 | ± | 6.49 | 23.70 | ± | 6.81 | 25.38 | ± | 7.02 |
| 14-oxo-MaR1 | 0.05 | ± | 0.05 | 0.10 | ± | 0.11 | 6.20 | ± | 2.80* | 0.00 | ± | 0.00 | 0.00 | ± | 0.00 | 6.49 | ± | 3.43* |
| 7S,14S diHDHA | 88.95 | ± | 29.33 | 82.33 | ± | 26.34 | 49.74 | ± | 17.26 | 94.35 | ± | 26.19 | 80.49 | ± | 23.80 | 54.24 | ± | 14.37 |
| 4,14-diHDHA | 37.45 | ± | 9.74 | 33.45 | ± | 6.26 | 34.51 | ± | 8.26 | 47.16 | ± | 12.96 | 39.03 | ± | 11.00 | 42.50 | ± | 12.25 |
| **n-3 DPA bioactive metabolome** | | | | | | | | | | | | | | | | | | |
| RvT1 | 1.47 | ± | 0.63 | 0.77 | ± | 0.41 | 0.57 | ± | 0.27 | 0.93 | ± | 0.41 | 0.62 | ± | 0.43 | 2.30 | ± | 1.95 |
| RvT2 | 0.00 | ± | 0.00 | 0.00 | ± | 0.00 | 0.08 | ± | 0.08 | 0.00 | ± | 0.00 | 0.00 | ± | 0.00 | 0.00 | ± | 0.00 |
| RvT3 | 0.00 | ± | 0.00 | 0.00 | ± | 0.00 | 0.00 | ± | 0.00 | 0.00 | ± | 0.00 | 0.00 | ± | 0.00 | 0.00 | ± | 0.00 |
| RvT4 | 24.18 | ± | 7.02 | 19.22 | ± | 5.99 | 17.56 | ± | 5.77 | 24.21 | ± | 6.95 | 23.91 | ± | 8.32 | 26.24 | ± | 9.27 |
| RvD1_n3 DPA_ | 10.81 | ± | 2.24 | 10.98 | ± | 2.56 | 11.41 | ± | 2.47 | 9.92 | ± | 2.10 | 10.40 | ± | 2.73 | 10.12 | ± | 2.64 |
| RvD2_n3 DPA_ | 3.72 | ± | 0.72 | 4.32 | ± | 0.63 | 3.38 | ± | 1.03 | 3.29 | ± | 0.68 | 2.69 | ± | 0.75 | 2.38 | ± | 0.88 |
| RvD5_n3 DPA_ | 47.16 | ± | 13.29 | 39.13 | ± | 10.60 | 36.85 | ± | 9.67 | 51.73 | ± | 13.66 | 51.39 | ± | 13.72 | 49.76 | ± | 14.68 |
| PD1_n3 DPA_ | 0.49 | ± | 0.49 | 0.38 | ± | 0.43 | 1.32 | ± | 0.98 | 0.12 | ± | 0.12 | 0.00 | ± | 0.00 | 0.00 | ± | 0.00 |
| 10S,17S-diHDPA | 32.14 | ± | 10.52 | 29.10 | ± | 9.10 | 27.05 | ± | 9.62 | 39.63 | ± | 13.23 | 33.70 | ± | 11.26 | 37.77 | ± | 13.55 |
| MaR1n3 DPA | 0.00 | ± | 0.00 | 0.00 | ± | 0.00 | 10.07 | ± | 7.20 | 0.00 | ± | 0.00 | 0.00 | ± | 0.00 | 0.75 | ± | 0.84 |
| 7S,14S-diHDPA | 96.27 | ± | 33.81 | 84.82 | ± | 29.63 | 66.34 | ± | 28.25 | 105.37 | ± | 29.41 | 94.60 | ± | 28.17 | 86.37 | ± | 29.06 |
| **EPA bioactive metabolome** | | | | | | | | | | | | | | | | | | |
| RvE1 | 0.00 | ± | 0.00 | 0.00 | ± | 0.00 | 0.00 | ± | 0.00 | 0.00 | ± | 0.00 | 0.00 | ± | 0.00 | 0.00 | ± | 0.00 |
| RvE2 | 70.66 | ± | 15.55 | 79.57 | ± | 19.96 | 73.07 | ± | 18.58 | 79.33 | ± | 20.07 | 75.97 | ± | 19.10 | 75.12 | ± | 21.47 |
| RvE3 | 6.75 | ± | 1.56 | 5.71 | ± | 1.36 | 5.50 | ± | 1.44 | 6.52 | ± | 1.60 | 4.98 | ± | 1.36 | 6.75 | ± | 1.80 |
| **AA bioactive metabolome** | | | | | | | | | | | | | | | | | | |
| LXA_4_ | 4.27 | ± | 0.78 | 37.74 | ± | 19.88 | 3.82 | ± | 0.84 | 4.27 | ± | 1.00 | 35.32 | ± | 17.43 | 4.05 | ± | 1.27 |
| LXB_4_ | 2.64 | ± | 1.62 | 2.74 | ± | 1.93 | 3.56 | ± | 2.44 | 3.85 | ± | 2.41 | 2.41 | ± | 2.70 | 6.12 | ± | 3.63 |
| 5,15-diHETE | 3143.64 | ± | 983.41 | 2983.39 | ± | 1001.02 | 3034.08 | ± | 1042.25 | 3589.82 | ± | 1061.76 | 3344.14 | ± | 1047.79 | 3504.40 | ± | 1123.62 |
| 15-epi-LXA_4_ | 70.05 | ± | 4.76 | 450.50 | ± | 36.53 | 88.78 | ± | 3.31* | 73.31 | ± | 6.81 | 529.01 | ± | 158.59 | 70.26 | ± | 9.92 |
| 15-epi-LXB_4_ | 10.73 | ± | 1.40 | 10.11 | ± | 1.63 | 6.81 | ± | 2.01 | 10.93 | ± | 0.92 | 10.63 | ± | 3.44 | 9.98 | ± | 0.75 |
| 13,14-dihydro-15-oxo-LXA_4_ | 15.47 | ± | 0.46 | 17.17 | ± | 1.76 | 16.54 | ± | 0.99 | 16.31 | ± | 1.08 | 17.08 | ± | 1.06 | 15.66 |  | 1.82 |
| 15-oxo-LXA_4_ | 13.94 | ± | 8.62 | 15.87 | ± | 11.23 | 17.29 | ± | 11.91 | 15.52 | ± | 9.54 | 17.78 |  | 12.18 | 18.71 |  | 12.81 |
| LTB_4_ | 0.00 | ± | 0.00 | 0.00 | ± | 0.00 | 0.00 | ± | 0.00 | 0.00 | ± | 0.00 | 0.00 | ± | 0.00 | 0.00 | ± | 0.00 |
| 5,12 diHETE | 488.62 | ± | 147.74 | 499.27 | ± | 170.85 | 435.47 | ± | 138.13 | 540.91 | ± | 139.31 | 476.29 | ± | 129.77 | 546.62 | ± | 162.94 |
| 6-trans-LTB_4_ | 235.92 | ± | 68.87 | 223.47 | ± | 72.35 | 223.07 | ± | 69.21 | 271.31 | ± | 80.25 | 260.65 | ± | 91.17 | 272.32 | ± | 92.32 |
| 12-epi-6-trans-LTB_4_ | 233.43 | ± | 73.23 | 233.84 | ± | 79.92 | 234.06 | ± | 78.53 | 251.86 | ± | 73.16 | 237.43 | ± | 79.45 | 257.11 | ± | 87.64 |
| PGD_2_ | 328.65 | ± | 78.25 | 427.15 | ± | 111.45* | 349.30 | ± | 55.57 | 434.23 | ± | 81.17 | 391.42 | ± | 71.36 | 390.64 | ± | 84.92 |
| PGE_2_ | 2209.17 | ± | 486.38 | 2139.01 | ± | 570.51 | 2302.43 | ± | 703.01 | 3426.89 | ± | 948.93 | 2816.05 | ± | 840.18* | 3033.88 | ± | 901.60* |
| PGF_2α_ | 70.16 | ± | 20.81 | 82.26 | ± | 22.87 | 93.25 | ± | 26.26 | 83.60 | ± | 26.21 | 58.52 | ± | 22.71* | 72.12 | ± | 25.31 |
| TXB_2_ | 13.39 | ± | 3.18 | 15.62 | ± | 4.91 | 14.57 | ± | 3.94 | 12.78 | ± | 3.26 | 11.49 |  | 2.32 | 14.24 | ± | 3.11 |

Tendon stromal cells (60,000 cells per well) were derived from Achilles tendinopathy (AT n=5 donors) or Achilles rupture (AR n=5 donors) and incubated in the presence of 10nM 15-epi-LXA_4_, 10nM MaR1 or vehicle only in the presence of 10ngml^-1^ IL1β. Cell incubations were terminated using ice-cold methanol containing internal standards and lipid mediators (LM) were identified and quantified using LM-profiling (see methods for details). Results are expressed as pg/incubation. Mean ± SEM of n = 9 per incubation. * p ≤ 0.05 comparison between respective vehicle incubated tendon stromal cells. The detection limit was ~ 0.1 pg. 0.0, Below limits of detection.
